# Parents’ Experiences From Participating in an Online Peer Support Child Feeding Intervention: The “PICNIC Sweden” Explanatory Sequential Mixed Methods Feasibility Study

**DOI:** 10.2196/93565

**Published:** 2026-09-22

**Authors:** Kerith Duncanson, Anna Jörnvi, Sara Ask, Malin Högman, Erica Belestam Vikman, Richard Ball, Maria Henström Engblom

**Affiliations:** 1 School of Medicine and Public Health, College of Health, Medicine and Wellbeing, University of Newcastle Australia, Callaghan, New South Wales, Australia; 2 Nutrition and Metabolic Health Research Program, Hunter Medical Research Institute, New Lambton Heights, New South Wales, Australia; 3 Department of Medicine, Huddinge, Karolinska Institutet, Blickagången 16, Huddinge, Stockholm, 141 83, Sweden; 4 Sara Ask Produktion AB, Stockholm, Sweden; 5 Health Promotion, Mid North Coast Local Health District, Port Macquarie, New South Wales, Australia; 6 School of Health Sciences, College of Health, Medicine and Wellbeing, University of Newcastle Australia, Callaghan, New South Wales, Australia

**Keywords:** child nutrition, child health, child feeding, responsive feeding, parent, primary prevention, feasibility studies, peer support, peer education, child health services

## Abstract

**Background:**

Parents’ feeding practices play a role in shaping children’s dietary patterns, which influence chronic disease risk. Feeding children is challenging, so parents need guidance and support. Parents In Child Nutrition Informing Community (PICNIC) is an Australian program that promotes responsive child feeding using dietitian-led training and online communication, then facilitates peer-to-peer information sharing within parents’ social networks.

**Objective:**

This pilot study aimed to test the feasibility, acceptability, appropriateness, and perceived impact of PICNIC with Swedish parents.

**Methods:**

This explanatory sequential mixed methods study involved 47 parents of children aged 0 to 24 months. Parents attended a 2-hour online dietitian-led workshop, followed by 2 months of child nutrition and feeding support in a moderated, closed Facebook group. Quantitative data from digital questionnaires (n=43) were analyzed and integrated with qualitative findings from thematic analysis of semistructured interviews (n=15).

**Results:**

Parents attended 1 of 10 workshops, and 4 to 5 posts per week (of which 20% were participants’ questions) were shared in the project’s Facebook group. Both quantitative and qualitative data indicated parents’ child feeding knowledge and parental self-efficacy increased after participation. Parents reported that the intervention filled a gap in the existing support from child health services, strengthening their confidence in child feeding, while providing a sense of recognition and community. PICNIC information was described as trustworthy, shareable, and something to “lean on.” Parents valued the social interaction during the workshops but also suggested providing recorded content for easier access.

**Conclusions:**

This pilot study demonstrated high acceptability and appropriateness of the PICNIC intervention model among parents in Sweden. This study will inform the design of an adapted version of PICNIC to be offered through child health services.

## Introduction

### Background

Promotion of healthy lifestyle habits in early childhood is of paramount importance [1], as eating behaviors and dietary patterns develop early in life [2] and often persist into adulthood [3]. Children’s eating behaviors are influenced by their parents’ food habits and feeding strategies [4,5]. Responsive feeding is an evidence-based approach in which parents recognize and respond appropriately to children’s hunger and satiety cues, while also modeling healthy eating behaviors [6]. It emphasizes repeated, neutral (nonpressured) exposure to a variety of foods, which has been shown to shape children’s preferences [7], such as increasing fruit and vegetable consumption [8].

Parents often describe child feeding issues as stressful and can find it difficult to translate available information into practice [9,10]. Qualitative research has shown that parents’ child feeding practices are influenced more by peers (family and friends) than by dietary guidelines [10]. In addition, parents increasingly seek information and encounter advertising on social media and the internet, and may therefore be exposed to a confusing array of misinformation and conflicting advice as they strive to feed their children optimally [9]. Normal child eating behaviors, such as neophobia or “fussy or picky eating”, also create concerns for parents [11,12] and can lead to nonresponsive parenting strategies such as pressure to eat or offering only accepted “safe foods,” possibly impacting negatively on children’s food preferences and development [13,14]. Higher perceived parenting competence, such as self-efficacy and satisfaction, has been reported to be positively associated with greater use of responsive food parenting practices [15].

In Sweden, the child health services (CHS) are responsible for offering parenting and child feeding support, aiming to empower parents through conversations during regular health visits and parent group sessions at the CHS center [16]. This health promotion work is particularly important, considering that less than 1 in 10 children and adolescents in Sweden meet the recommended fruit and vegetable intake [17,18] and approximately 10% of children have developed overweight by 4 years of age [19]. However, the national CHS program is multifaceted, leaving limited time for individualized, nutrition-related discussions [20]. CHS nurses frequently identify time constraints as a barrier to addressing family lifestyle habits, and these are complex topics that can be perceived as both sensitive and challenging to talk about [21,22]. Thus, there is a need for evidence-based resources to facilitate the support.

The “Parents In Child Nutrition Informing Community” (PICNIC) program is an ongoing population-level nutrition intervention in the Mid North Coast Local Health District, Australia, aimed at improving parental feeding practices and children’s dietary quality [23]. PICNIC parents are trained in dietitian-led workshops and provided with ongoing, evidence-based plain-language child feeding information through online resources (website, Facebook, and Instagram; Meta Platforms, Inc). This peer education model was informed by formative research, which recommended early recruitment (<6 mo) and easy access to shareable online child feeding information to facilitate knowledge exchange and peer support between parents [10,24,25]. Parents co-designed the intervention, actively shared information with peers, contributed their experiences, and provided feedback to the research team through participatory action research [26]. Parents accessing PICNIC have shown improvement in child feeding practices and higher intake of vegetables in children compared to age-matched population data (Ball et al, manuscript submitted), and content analysis indicates that parents engage most with instructive (“how-to”) content and messages on food restriction and fussy eating [27]. The online shareable format has shown potential to reach more than those directly engaged in the program [28]. For example, while a total of 1250 parents have been trained in a PICNIC workshop to date, PICNIC social media content reached almost 17,000 unique users on Facebook over the last month (as of January 2026; insights from Meta Business Suite).

The PICNIC project has attracted great interest from child and family health centers and is currently being scaled to other Australian health districts and new settings. This peer-to-peer education model may also be a suitable strategy to influence parents’ feeding practices in Swedish CHS where no comparable programs currently exist. A feasibility trial is warranted to evaluate PICNIC transferability to the Swedish context and ensure that the program is tailored to meet end users’ needs and expectations.

### Aim

This pilot study aimed to assess the feasibility, acceptability, appropriateness, and perceived impact of the online peer education child feeding program PICNIC, developed in Australia, to be offered to parents of young children in Sweden.

## Methods

### Study Design

“PICNIC Sweden” was a mixed-methods single-group feasibility study designed to let parents in Sweden test an adapted version of the PICNIC child feeding peer education program, originally developed in Australia [23,26]. We used an explanatory sequential design integrating both quantitative and qualitative data to gain a comprehensive understanding of various feasibility aspects of the intervention in Sweden [29]. Data were collected concurrently between October 2023 and February 2024 and consisted of digital questionnaires, social media analytics, and semistructured interviews. This study is reported according to the COREQ (Consolidated Criteria for Reporting Qualitative Research) checklist (qualitative interviews) [30] and the guidelines for Good Reporting of A Mixed Methods Study (GRAMMS) [31] (Checklist 1).

### Participants and Recruitment

Participants in this study were parents or primary caregivers of a child aged 0 to 24 months. To participate, parents had to be aged at least 18 years, reside in Sweden, and understand written and spoken Swedish sufficiently well to participate in the workshop and answer the surveys. Recruitment took place between October 1 and November 15, 2023, using convenience sampling in the health care region of Sörmland, combined with snowball (peer-to-peer) sampling. All 26 child health centers located in the region were sent recruitment material, including posters and brochures, for display and distribution within their centers and social media post templates for online CHS pages and groups. A link referred to the recruitment webpage at ki.se and the expression of interest form. Interested parents were emailed full study information and offered the option to receive further information via phone before consenting to participate in the study.

### PICNIC Intervention

**Figure 1.**
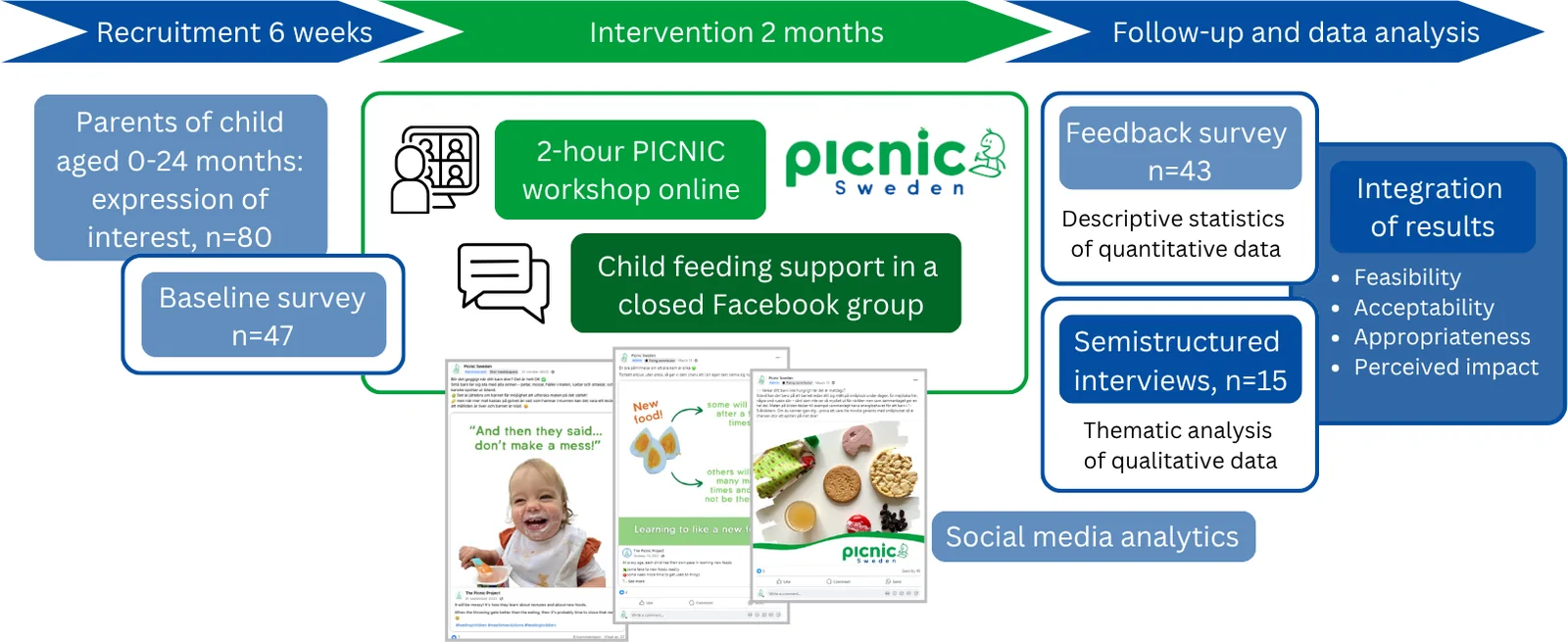
Study design with flow of participants, data collection and analysis, and the “PICNIC Sweden” intervention with examples of Facebook content.

The intervention consisted of online training (a 2-hour virtual workshop on Zoom [Zoom Communications, Inc]) on food introduction and responsive feeding practices, as well as social network support resources (Figure 1). A registered dietitian (SA) with extensive experience working with families led the workshop, which was adapted from the Australian PICNIC project to a Swedish context. Thus, the same food introduction and responsive feeding concepts were covered, while the content was translated and adapted to Swedish dietary guidelines and the local food context. The only structural changes compared to the Australian original intervention included virtual workshops only (no face-to-face sessions) and no follow-up sessions, while the workshop duration remained unchanged (2 h). The participants were also invited to a closed Facebook group managed by the research team. In this forum, approximately 3 posts per week were shared to reinforce PICNIC messaging, either reshared from the Australian PICNIC page with Swedish captions or created by MHE and SA. The posts covered the same key concepts of responsive child feeding as those in the original Australian PICNIC and used a similar messaging tone. Parents could also submit child feeding questions through user-created posts, which were approved by the group administrator before being published and answered by the dietitian (SA) for the benefit of all group members. Group members could react (“like”) and comment on all posts but could not share them outside the group due to Facebook privacy settings. As PICNIC used a peer education approach, participants were allowed to invite their partner, family, and parent friends to the Facebook group. However, entry required agreement to the group rules, which clearly outlined the group’s purpose as part of a research project and explained that group-level engagement metrics would be collected. For this pilot study, no public resources were developed; only the closed Facebook group was used. Participants were, however, encouraged to visit the Australian PICNIC project website and social media pages (Facebook and Instagram) for additional resources.

### Data Collection

#### Questionnaires

A secure digital platform (REDCap, hosted at Karolinska Institutet) was used to distribute consent forms and digital questionnaires. A baseline questionnaire that included background parent or family and child characteristics and questions about previous experience of parent groups, perceived self-efficacy, and information-seeking behavior about healthy food and child feeding was distributed by email before participants attended the workshop. Two months after workshop participation, each of the 47 parents was approached via email and asked to fill in a follow-up questionnaire evaluating their program experience and providing feedback with regard to the research questions (feasibility, acceptability, appropriateness, and perceived impact). The questions were developed based on previous feasibility and intervention studies [24,32,33] and single items in validated questionnaires [34,35], modified to fit the current intervention, setting, and research aims. The questionnaires were pilot tested within the research team to ensure usability prior to data collection.

#### Social Media Analytics

Basic metrics, including the number of members as well as “active” engagement such as reactions or “likes” and comments, were exported from the Facebook group to Microsoft Excel by the administrator MHE. The data were then manually verified and supplemented with additional details, including post views (a type of “silent” engagement) by AJ after the study period. Only post reactions made by study participants were counted and included in the summary.

#### Semistructured Interviews

Semistructured individual interviews were conducted 2 to 3 months after the workshop (January to February 2024). The aim was to gain a deeper understanding of parents’ views and experiences of the intervention, including perceived barriers to and facilitators of engaging in it, and potential impact. Using maximum variation sampling, 20 participants were invited in the first round. The study sample was subdivided by education level, country of birth, gender, number of children, and age or gender of child for random selection by characteristic groupings. Nine parents consented to interviews (the reasons for nonparticipation were unknown). To reach the target of 15 interviews, which was considered adequate for acquiring rich and sufficiently detailed data, another 2 rounds of invitations were sent out to 6 and 3 additional participants, respectively, of whom 6 consented and completed an interview. Subsequent rounds on invitations were selected from those with the same combination of characteristics to increase the likelihood of capturing diverse perspectives and experiences. We intentionally sought to interview both mothers and fathers with varying educational levels and country of birth, as well as parents with one or multiple children, including both girls and boys across the age range represented in our study. Interviews were conducted via phone in Swedish by AJ, a female registered dietitian with clinical experience in counseling families with children and employed as a part-time research assistant in this project. The interviews were attended by AJ, the interviewee, and, in 2 cases, the interviewee’s partner. The interviewees were aware that AJ was a research assistant, but no other prior relationship existed. The interview followed a semistructured format with a preset interview guide (Multimedia Appendix 1) developed by AJ and MHE (project leader, nutritionist, and assistant professor experienced in early intervention). The interview guide was pilot tested with a mother of a 6-month-old girl (not included in the study), resulting in minor revisions. All interviews were audio recorded and transcribed verbatim using a professional transcribing service, with final transcripts not reviewed by participants. Interviews lasted a mean of 41 (SD 8, range 28-60) minutes.

### Data Analyses

#### Quantitative Analysis

The quantitative data in questionnaires as well as social media analytics were summarized with descriptive statistics using R (version 4.3.0; R Foundation for Statistical Computing) in RStudio (version 2023.3.0.386). A question on parental self-efficacy (PSE) for promoting healthy eating behaviors in their children, from the validated PSEPAD (Parental Self-Efficacy for Promoting Healthy Physical Activity and Dietary Behaviors in Children) instrument [35], was included in the questionnaires to enable comparison with previous studies and explore its distribution and change in this study population. The item response was a Likert-type scale, ranging from 0 (*not at all*) to 10 (*to a very high degree*). Although PSE change scores deviated from normality (Shapiro-Wilk test: *W*=0.90; *P*=.002), a 2-tailed paired *t* test was used as the primary analysis because of its robustness in samples of this size. A Wilcoxon signed-rank test was also conducted as a sensitivity analysis.

#### Qualitative Analysis

The 15 interview transcripts were analyzed using thematic analysis with an inductive approach [36,37]. First, data familiarization was performed through a read-through of all transcripts and initial discussions between AJ (registered dietitian), MHE, MH, and EBV, all female. Initial notes were taken to capture first impressions and emerging patterns in the data. MH and EBV were final-year bachelor’s students in nutrition at the time, working on the data as part of their degree project. Supervised by MHE, systematic coding was performed independently by AJ and MH using meaning units and codes in Microsoft Excel 2023 (version 16.78.3). To achieve a shared understanding, the individual coding results and preliminary themes were discussed between AJ, MH, MHE, and KD in an iterative process. The themes were also refined by MHE through a revisiting of transcripts, coded data, and notes from the initial discussions. Final theme names and their scope were reviewed by all authors to reach consensus. The preliminary analysis was carried out in Swedish, with themes and quotations translated into English for co-analysis by English-speaking authors and for publication.

#### Data Integration

Quantitative and qualitative data in this sequential mixed methods study [29] were integrated using side-by-side comparison within framework analysis to interpret findings. Framework domains reflected the aims of understanding feasibility, acceptability, appropriateness, and perceived impact of the PICNIC program.

### Ethical Considerations

All participants provided informed consent before participating in the study. To enter the closed Facebook group, members were required to accept the “group rules,” which clearly described the purpose of the Facebook group and that group-level engagement metrics would be collected and reported in scientific communications. For the interviewees, additional informed consent was obtained after they had received individual invitations and study information related to the interview. No compensation was given to participants. The study was conducted in accordance with the Declaration of Helsinki, and the Swedish Ethical Review Authority approved this study (Dnr 2023-04381-01).

## Results

### Characteristics of Participating Parents at Baseline

Over a 6-week period, 80 parents submitted an expression of interest (Figure 1). Of these, 47 (59%) parents gave formal study consent, filled in the baseline questionnaire, and were able to attend 1 of the 10 workshops held during the trial period. Twenty (43%) participants resided in Sörmland, and 27 (57%) were from 6 other neighboring health care regions across central Sweden. They reported being recruited through their child health center or nurse (n=15, 32%), a social media post (n=8, 17%), or a friend or family member (n=24, 51%). Table 1 presents descriptive characteristics of the participating parents. The majority were mothers (46/47, 98%) born in Sweden (44/47, 94%) and aged approximately 33.8 (SD 4.8; range 22‐44) years, and 43 (91%) participants had a university degree. Most participants had a child aged 6 to 12 months (22/47, 47%) or 0 to 6 months (17/47, 36%). While 25 (53%) parents had attended CHS parent groups, 20 (43%) reported not being invited to one at their child health center. The PSE was, on average, 7.8 (SD 1.5; range 5‐10). Before engaging in the PICNIC intervention, more than half (27/47, 58%) of participants replied they *quite often* or *very often* had questions on children’s food or feeding, and one-third (14/47, 30%) felt they had not received sufficient child feeding support from CHS. The most common sources of information were the internet, the Swedish Food Agency website, family, friends, or other peers. The 15 interviewed parents were comparable to the study population in terms of demographic and baseline characteristics.

**Table 1.** Characteristics of parents participating in the PICNIC^a^ Sweden pilot study.

Characteristics	All (n=47)	Interviewed (n=15)
Gender, n (%)		
Female	46 (98)	14 (93)
Male	1 (2)	1 (7)
Age (years), mean (SD)	33.8 (4.8)	32.8 (3.5)
Country of birth^b^, n (%)		
Sweden	44 (94)	13 (87)
Other	3 (6)	2 (13)
Education, n (%)		
University	43 (91)	13 (87)
No university	4 (9)	2 (13)
Number of children^c^, n (%)		
One child	41 (87)	11 (73)
Two children	4 (9)	3 (20)
Three children	2 (4)	1 (7)
Age of youngest child (months), mean (SD)	7.9 (4.2)	8.1 (4.9)
0‐6 months, n (%)	17 (36)	6 (40)
6‐12 months, n (%)	22 (47)	6 (40)
>12 months, n (%)	8 (17)	3 (20)
Gender of youngest child, n (%)		
Girl	10 (21)	4 (27)
Boy	30 (64)	10 (67)
One boy and one girl^c^	1 (2)	1 (6)
NA^d^	6 (13)	—^e^
How did you find out about the “PICNIC Sweden” project?, n (%)		
Child health center or nurse	15 (32)	8 (53)
A friend or family	24 (51)	6 (40)
Social media	8 (17)	1 (7)
Internet, Googled, or other	0 (0)	0 (0)
Have you participated in a CHS^f^ parent group with your child?, n (%)		
Yes, several times	16 (34)	3 (20)
Yes, one session	9 (19)	3 (20)
No, but the other parent has	0 (0)	0 (0)
No, didn’t want to/couldn’t	2 (4)	1 (7)
No, have not been invited	20 (43)	8 (53)
Do you usually take part in other parent groups outside of the child health center?^g^, n (%)		
Open preschools (*swe* öppen förskola)	30 (64)	10 (67)
Maternal health parent group	0 (0)	0 (0)
Friends	26 (55)	7 (47)
Social media (eg, groups)	22 (47)	4 (27)
Other	2 (4)	1 (7)
No, I only participate in parent groups at the child health center	4 (9)	1 (7)
No, I do not participate in parent groups (at child health center or outside it)	4 (9)	1 (7)
How confident are you that you can promote healthy eating habits for your child?		
Average PSE^h^ score (scale 0‐10), mean (SD)	7.8 (1.5)^i^	8.1 (1.4)
Do not know, n (%)	1 (0)	0 (0)
Do you tend to have questions or wonder about children’s food/feeding?, n (%)		
Very often (every day)	4 (9)	1 (7)
Quite often	23 (49)	6 (40)
Sometimes	17 (36)	7 (47)
Seldom	3 (6)	1 (7)
Never	0 (0)	0 (0)
Where do you turn to for information or answers to questions about children and food/feeding? *Answers coded as 1 (never), 2 (rarely), 3 (sometimes), 4 (often), and 5 (very often*), mean (SD)		
Internet (eg, Googling)	3.9 (1.0)	3.5 (1.1)
Social media	3.0 (1.3)	3.0 (1.0)
Friend and social contacts	3.3 (1.0)	3.3 (1.0)
Family	3.0 (1.1)	3.1 (1.0)
Child health center or nurse	3.2 (1.0)	3.3 (1.1)
Swedish Food Agency (website)	3.4 (1.0)	3.5 (1.1)
1177.se (national health care service)	3.1 (1.0)	3.2 (0.8)
Registered dietitian (eg, in health care)	1.4 (1.0)	1.5 (1.1)
Media (newspapers, radio, and TV)	1.6 (0.7)^i^	1.7 (0.7)^j^
If you get information or advice about children and food on social media, which platforms do you usually get it from?, n (%)		
Facebook	22 (47)	7 (47)
Instagram	31 (66)	12 (80)
TikTok	3 (6)	0 (0)
YouTube	1 (2)	1 (7)
Other platform	1 (2)	0 (0)
Do not get info from social media	3 (6)	0 (0)
Do not use social media	3 (6)	0 (0)
How much do you trust information about children and food that you find online? *Scale answers coded from 1 (do not trust at all) to 5 (have a lot of trust*), mean (SD)		
Trust score	3.1 (0.7)	3.2 (0.7)
Do you feel you have received sufficient information and support from CHS on child feeding?, n (%)		
Yes	21 (45)	8 (53)
No	14 (30)	4 (27)
Do not know	12 (25)	3 (20)

a PICNIC: Parents In Child Nutrition Informing Community.

b All participants understood written and spoken Swedish and English (OK or well).

c One participant who had 3 children was a mother of twins (a girl and a boy).

d NA: Not applicable (missing data).

e Missing data are reported as not applicable (NA).

f CHS: child health services.

g Multiple answers could be chosen; % may add up to more than 100%.

h PSE: parental self-efficacy.

i n=46.

j n=14.

### Process Evaluation and Social Media Engagement Metrics

Ten online workshops were held over a period of 5 weeks (October 10 to November 17, 2023), with an average of 4 to 5 participants per workshop, totaling 47 parents. An additional 3 fathers (not included in data collection) were present and listened in together with their participating partner. After the workshops, the closed Facebook group had grown to 60 members, including PICNIC team members or administrators, study participants, and peer-invited partners or friends. During the 4 months of Facebook group activities (October to January), a total of 52 intervention posts (including 9 video posts) were shared by the PICNIC Sweden team, and 17 user-created posts from parents were published and answered in the group. Regarding feeding-related messages, posts focused on children’s learning to eat process (n=10), division of responsibility, including pressure to eat and feeding cues (n=9), meal environment and role modeling (n=8), what or how to serve (n=8), fussy eating (n=7), food exposure (n=6), and other or mixed topics (n=4). Most videos (5/9) addressed learning to eat. Notably, many posts were multimessaged, reflecting the intertwined nature of responsive child feeding practices. A variety of communication techniques were used, such as “how-to” content, storytelling, rhetorical questions, relatable and realistic photos and videos, and real-world tie-ins (eg, Christmas or Easter food) [27]. In general, the active engagement in the group was low; posts received an average of 2 “likes” (minimum to maximum: 0‐7) from parents participating in the project. User-created posts always received some comments (3 on average, including the PICNIC dietitians’ answers to questions), whereas 8 (15%) of 52 PICNIC-created posts received comments from parents. However, silent engagement, that is, viewing or reading posts, was overall higher, and while PICNIC-shared posts received 39 views on average, user-created posts received 47 views, suggesting higher “silent” engagement from parents with these question-and-answer type of posts. The most viewed PICNIC Facebook content (>55 views) included posts on what foods to serve, when and how to serve water, family meals (eating together), and the concept of “parents provide, children decide.” The most engaging user-created post (58 views, 10 comments) was a parent’s question on the use of taste buttons to introduce babies to new flavors.

### Quantitative Survey Data—Follow-Up After PICNIC

Participants’ responses to the follow-up questionnaire are presented in Table 2 (n=43, 91.5% response rate). Overall, the parents reported high satisfaction with the intervention and seemed to find the workshop especially useful. They also reported gained knowledge or insights, especially around how to practically manage different mealtime situations with their children. The mean PSE scores increased from 7.69 (SD 1.52) at baseline to 8.17 (SD 1.40) at follow-up among participants with data at both time points (n=42). A paired *t* test showed that PSE scores were significantly higher at follow-up than at baseline, with a mean increase of 0.48 points (95% CI 0.11-0.84; *t*_41_=2.63; *P*=.01). A Wilcoxon signed-rank test yielded the same conclusion (*V*=601.5; *P*=.02). Furthermore, Facebook and Instagram were considered appropriate platforms for PICNIC, where open-ended answers (16/43, 37% chose to add a comment) explained that these were the most used platforms in this group today and the group function that Facebook provides was suitable for sharing in PICNIC; however, considering younger generations are not using Facebook as much, other platforms should be considered. Moreover, online engagement varied; while one-third (14/43, 33%) had actively viewed group posts 2 to 3 times per week and another third (15/43, 35%) more often than that, the remaining 32% (14/43) had viewed posts only once a week or more seldom (Table 2). Parents had shared information or advice from PICNIC primarily with the child’s other parent (33/43, 77%), friends (25/43, 58%), and/or family (24/43, 56%), and most (30/43, 70%) considered it appropriate to be invited to PICNIC when the child was 3 to 4 months of age. Parents’ opinions on the preferred format of the PICNIC workshop varied; most favored the online workshop as it was (41/43, 95%), but in-person meetings (14/43, 33%) and prerecorded information (17/43, 40%) were also suggested. Open answers to this question (28/43, 65% chose to comment) identified both pros and cons with different modes of delivery. Live workshops were considered valuable as questions could be asked directly to the dietitian. Parents commented that in-person meetings could be more socially enjoyable and boost discussions; however, getting there with the child may be challenging and time-consuming. Attending from home or from “anywhere” was considered more feasible. Prerecorded or self-directed options were commented upon as a good option for those who could not attend at a specific time or who wanted to relisten but were suggested as a complement combined with interactive sessions or workshops to gain the benefits from both.

**Table 2.** Participants’ response to the follow-up survey after 2 months of engagement with the PICNIC^a^ Sweden child feeding support (n=43).

Question or statement and response	Values
Part 1. The dietitian-led online PICNIC workshop, n (%)	
To what extent do you agree with these statements?	
The workshop gave me useful tips/advice/information	
Strongly disagree	—^b^
Disagree	—
Neither agree nor disagree	—
Agree	12 (28)
Strongly agree	31 (72)
Participating online (on Zoom) worked well	
Strongly disagree	—
Disagree	—
Neither agree nor disagree	—
Agree	9 (21)
Strongly agree	34 (79)
I liked participating in the PICNIC workshop	
Strongly disagree	—
Disagree	—
Neither agree nor disagree	1 (2)
Agree	8 (19)
Strongly agree	34 (79)
I would recommend the workshop to other parents	
Strongly disagree	—
Disagree	—
Neither agree nor disagree	1 (2)
Agree	9 (21)
Strongly agree	33 (77)
In what format do you think the PICNIC workshop should be?^c^^,^^d^ *Multiple responses allowed.*	
Online workshop (like we had)	41 (95)
In person (at the child health center or other venue)	14 (33)
Prerecorded (to watch at your own convenience)	17 (40)
Other way	—
Do not know	—
What do you consider a suitable duration for the workshop?	
Not more than 1 hour	1 (2)
1‐1.5 h	15 (35)
2 h (like we had)	23 (53)
2.5‐3 h	3 (7)
More than 3 h	—
Do not know	1
If you had been offered additional workshops, such as follow-up Q&A^e^ sessions online, would you have wanted to participate?	
Yes	34 (79)
No	—
Do not know	9
Part 2. Other parts of PICNIC, n (%)	
Other than the workshop, what other parts of PICNIC have you engaged with?^c^	
The closed Facebook group “PICNIC Sweden”	41 (95)
The Australian PICNIC website (picnicproject.com.au)	6 (14)
The Australian Facebook page (“The PICNIC Project”)	8 (19)
The Australian PICNIC Instagram (@picnic_nsw)	10 (23)
None of these (participated in the workshop only)	2 (5)
What do you think about the closed Facebook group “PICNIC Sweden” and the information shared there?	
The Facebook group gave me useful tips/advice/information	
Strongly disagree	—
Disagree	—
Neither agree nor disagree	5 (12)
Agree	19 (44)
Strongly agree	19 (44)
Social media is an appropriate channel for sharing child feeding information in PICNIC	
Strongly disagree	1 (2)
Disagree	—
Neither agree nor disagree	3 (7)
Agree	16 (37)
Strongly agree	23 (53)
I enjoyed being part of the Facebook group	
Strongly disagree	—
Disagree	—
Neither agree nor disagree	4 (9)
Agree	18 (42)
Strongly agree	21 (49)
I would recommend the closed Facebook group to other parents	
Strongly disagree	—
Disagree	2 (5)
Neither agree nor disagree	7 (16)
Agree	15 (35)
Strongly agree	19 (44)
How often have you read posts shared in the Facebook group? Think about a usual week during the period between joining the group and now (last 2 months).	
Several times per day	—
Once a day	5 (12)
More than 3 times per week	10 (23)
2‐3 times per week	14 (33)
Once a week	12 (28)
2‐3 times per month	1 (2)
Once a month or less	—
Never	1 (2)
Have you experienced any negative comments from others in the Facebook group?	
No	40 (93)
Yes	—
Do not know	3
Which social media platforms do you think PICNIC should be present on?^c^^,^^d^	
Facebook	40 (93)
Instagram	33 (77)
TikTok	3 (7)
Snapchat	1 (2)
X (formerly Twitter)	1 (2)
Other social media platform	1 (2)
None	1 (2)
Do not know	1
Would you like to continue being part of the closed Facebook group (PICNIC Sweden)?	
Yes	40 (93)
No	—
Do not know	3
Part 3. Experiences and perceptions of the PICNIC Sweden intervention as a whole	
What is your overall impression of PICNIC?, n (%)	
PICNIC is suitable for sharing information and support on child feeding	
Strongly disagree	—
Disagree	—
Neither agree nor disagree	—
Agree	8 (19)
Strongly agree	35 (81)
I perceive the information provided in PICNIC as factually correct	
Strongly disagree	—
Disagree	—
Neither agree nor disagree	—
Agree	7 (16)
Strongly agree	36 (84)
PICNIC answers questions that are common among parents of young children	
Strongly disagree	—
Disagree	—
Neither agree nor disagree	—
Agree	7 (16)
Strongly agree	36 (84)
I believe there is a need among parents in Sweden to have access to a project like PICNIC	
Strongly disagree	—
Disagree	—
Neither agree nor disagree	1 (2)
Agree	4 (9)
Strongly agree	38 (88)
How has your participation in PICNIC affected you? I have gained more knowledge/insights around:, n (%)	
what type of foods are healthy for my child	
Strongly disagree	—
Disagree	—
Neither agree nor disagree	5 (12)
Agree	19 (44)
Strongly agree	19 (44)
what amount of food is appropriate for my child	
Strongly disagree	—
Disagree	—
Neither agree nor disagree	7 (16)
Agree	18 (42)
Strongly agree	18 (42)
how I introduce new foods to my child	
Strongly disagree	—
Disagree	—
Neither agree nor disagree	3 (7)
Agree	11 (26)
Strongly agree	29 (67)
how I can practically manage different mealtime situations with my child	
Strongly disagree	—
Disagree	—
Neither agree nor disagree	1 (2)
Agree	13 (30)
Strongly agree	29 (67)
how I can respond to a child who doesn’t want to eat what is served	
Strongly disagree	—
Disagree	—
Neither agree nor disagree	1 (2)
Agree	14 (33)
Strongly agree	28 (65)
How confident are you that you can promote healthy eating habits for your child?, mean (SD)	
PSE score (scale 0‐10; n=43)	8.1 (1.4)
Have you shared information/tips/advice from PICNIC to others?¹ Verbally/in writing/digitally/on social media, n (%)	
The child’s other parent(s)	33 (77)
Friends	25 (58)
Family	24 (56)
Parent group/early childhood center	14 (33)
CHS nurse	6 (14)
Others	1 (2)
I have not shared information with others	2 (5)
Did you recommend others to participate in the “PICNIC Sweden” project?^c^, n (%)	
The child’s other parent(s)	10 (23)
Friends	28 (65)
Family	5 (12)
Parent group/early childhood center	15 (35)
Others	1 (2)
I have not actively recommended anyone	5 (12)
From what age of the child do you think one should be invited to PICNIC?, n (%)	
Already before birth (through maternal health clinic)	5 (12)
1 month/2 months	—
3 months	13 (30)
4 months	17 (40)
5 months	5 (12)
6 months	3 (7)
7/8/9/10/11/12 months or older^f^	—

a PICNIC: Parents In Child Nutrition Informing Community.

b No responses.

c Multiple responses allowed; percentages may exceed 100%.

d Respondents could also comment on their answers to these questions (optional).

e Q&A: question and answer.

f Response options with no answers from participants were grouped in this table.

#### Qualitative Insights From Interviews

**Figure 2.**
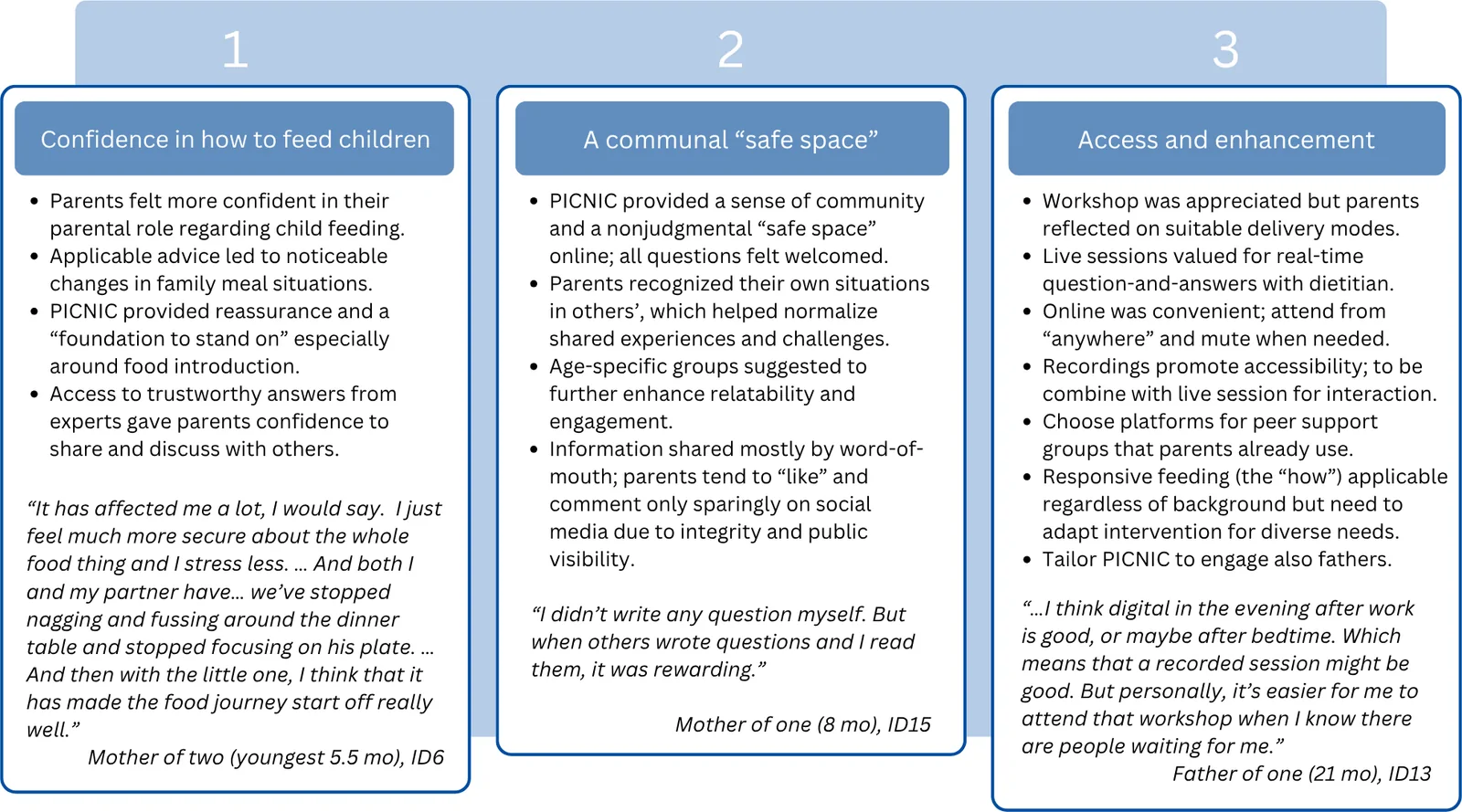
Themes in data from semistructured interviews with PICNIC (Parents In Child Nutrition Informing Community) participants.

Thematic analysis of transcripts from the 15 interviews (descriptive characteristics in Table 1) resulted in 3 themes. These are presented in Figure 2 and described below. Selected quotes have been labeled with an identifying number, *mother* or *father*, total number of children, as well as age of their youngest child when enrolling in the project.

#### Theme 1: Confidence in How to Feed Children

The parents described the intervention as pedagogic and useful. They reported increased understanding of how children learn to eat and perceived the information and advice as easy to absorb and apply in practice. This feeding efficacy led to increased self-confidence and greater trust in their child’s own ability during mealtimes. For instance, parents reported feeling less anxious about offering new foods and textures for their young children to explore:

*Not to get stuck with purées and ready-made baby food jars, but rather to cut up pieces of something easy to grip and gnaw on—and well, to dare a bit more.*

[Mother of one (6 mo), ID8]

It was evident during the interviews that parents had learned about responsive feeding; they spoke about the importance of creating curiosity and joy for food, being a role model, and maintaining a neutral expression toward the child’s eating behaviors and amount eaten. Many parents had become more aware of their own behaviors at mealtimes, and they shared stories on how they had changed practices and approach at home, resulting in a more positive atmosphere at the table:

*I don’t run around tidying up in the kitchen while he’s eating—instead, I sit with him.*

[Mother of two (youngest 6 mo), ID11]

Most participants emphasized that questions and concerns tend to arise when solid food is introduced and would be the ideal time to invite parents to PICNIC. Participating parents who had just entered their “food journey” also described the workshop as particularly reassuring, giving them “a foundation to stand on”:

*We were just at the beginning of our food journey with our daughter, and there was a sense of reassurance when hearing about, well, how to think about it, what needs to be considered, and what to stress over and not to stress over. I thought that felt super comforting.*

[Mother of one (7.5 mo), ID2]

Parents with previous feeding experience with older children also found the intervention useful. Some felt reassured knowing that their feeding approach aligned with current evidence, while others reported reflecting on their past practices and now adopted new feeding strategies when introducing food to their second child. This was described as motivated by encouragement rather than blame or judgment:

*But then when this project came along and everything...well, suddenly things just made sense. And became easier, I would say. I’d say that the food journey now with our second child has started off much better—easier.*

[Mother of two (youngest 5.5 mo), ID6]

Participants’ increased confidence and self-efficacy were partly attributable to their perception of the PICNIC content as trustworthy. Parents felt comfortable sharing PICNIC content because the advice was evidence-based and delivered and moderated by dietitians and nutritionists. Most had shared the information with partners, friends, or extended family members, who generally responded with interest and curiosity. Some also felt more comfortable discussing child feeding with their own parents:

*Then my mom said, “Can’t you just feed him, isn’t he too young to eat by himself?” That’s when I could use this information and explain that it’s evidence-based—there are benefits to letting him squeeze and feel the food, look at it, and learn to put it in his mouth.*

[Mother of one (5.5 mo), ID3]

Access to child feeding and nutrition expertise was recognized and highly valued by participants and also saved them time and effort relative to trying to access credible information online:

*It was really nice to be able to ask so many questions and have someone who is an expert answer them every time. I felt like there’s nowhere else you get that opportunity in the same way...I can search for as much information as I want, but to ask specific questions...felt very luxurious and...good.*

[Mother of one (7.5 mo), ID2]

At the same time, participants emphasized the importance of experts being relatable and nonjudgmental:

*She really seems very competent and good at talking to...people in an understandable way.*

[Mother of one (7 mo), ID12]

Overall, participation in PICNIC provided parents with the type of information and strategies they needed to transition toward responsive child feeding. This was evidenced by apparent, constructive change in the feeding dynamic with their child, and in parents’ reported feelings of reassurance and confidence, which motivated further engagement with the PICNIC online community and sharing of PICNIC principles with peers.

#### Theme 2: A Communal “Safe Space”

Being able to witness other parents’ questions was highly appreciated by the participants, both during the workshop and in the Facebook group. Parents described how they could recognize their own situation in others, which created a reassuring feeling and a sense of not being the only one feeling “a bit lost”:

*Sometimes there are things you haven’t even thought about yourself that other people bring up. And things you feel so alone in and think you’re the only one who has...those issues. But then you realize that others experience the same.*

[Mother of two (youngest 5.5 mo), ID6]

Several parents reported that participating in the project and being part of the supportive online group supported a sense of community, where hearing from other parents helped counter feelings of loneliness in their parental role. Thus, PICNIC filled a gap, particularly for those who had not attended parent groups at their child health center:

*At the child health centre I go to, there are no parent groups or anything. And food is a really big thing, especially in the beginning [of parenthood]. So, I have appreciated being able to get advice from other parents and, well, having the opportunity to engage in that way.*

[Mother of one (3.5 mo), ID4]

Some parents suggested additional workshops, with content tailored to the child’s age, including ages beyond 0 to 2 years, and with online parent groups organized accordingly. This was perceived as a way to enhance the relevance and engagement of the content, as it would be even easier for parents to relate to. However, one parent pointed out that it could also be beneficial for first-time parents to hear about the experiences of those with older children:

*At the same time, I think it can be helpful for first-time parents to hear from those who have more than one child, because as a first-time parent, you’re often more worried, cautious, and maybe a bit nervous about it.*

[Mother of two (youngest 4 mo), ID10]

Integral to the sense of community was the closed Facebook group being seen as a “safe space” to share. Parents appreciated its nonjudgmental tone and felt that all questions were welcomed, making it easier to engage:

*It’s a very friendly tone. Even I, who don’t usually ask or comment much, have done so. Just because I’ve felt that the environment has been very, well, pleasant.*

[Mother of three (youngest twins 8 mo), ID5]

While some parents actively engaged with PICNIC content or posted questions in the Facebook group, others preferred to observe. Although they found the content interesting and useful, they tended to comment or “like” only sparingly. Parents explained that this reflected personal considerations of privacy and a concern about being publicly visible and potentially judged, both within the group and on social media more broadly:

*It’s like...when you ask a question or post something, it’s as if you’re stepping out onto a stage...and in a way, exposing yourself.*

[Mother of one (7 mo), ID7]

Participants perceived that having attended the online workshop encouraged them to feel comfortable to share or comment on posts. Keeping the groups relatively small and local, as well as meeting each other in person, was believed to further enhance engagement:

*I think it also gives a sense of security, like, “Okay, I know who’s going to read this, I know them a little.”*

[Mother of one (16 mo), ID1]

Notably, while most content was consumed online, on-sharing was done primarily through word-of-mouth:

*I haven’t done it [shared content] on social media. But I’ve talked a lot about it.*

[Mother of one (7.5 mo), ID2]

Overall, parents responded positively to the peer education model, explaining that one typically perceives information as more trustworthy when obtained from someone with personal experience and that you know. Some parents described how friends who became parents found food introduction overwhelming and feared making mistakes, and PICNIC was seen as an opportunity to share a reassuring resource that friends could rely on.

*When mentioning this project, everyone has been really curious and kind of like, “We would’ve wanted this too.” So I think many people...well, sometimes you don’t realize you’re missing something until you have it, and it’s been a bit like that with PICNIC*.

[Mother of two (youngest 5.5 mo), ID6]

Participants emphasized the importance of involving fathers, recognizing their significant role in child feeding. The predominance of mothers in parent groups at child health centers and in child feeding groups on social media was reflected upon. Some participants attributed this to the common practice in Sweden of mothers staying home during the child’s first year, leading to a greater involvement in feeding. Others suggested that differences in information-seeking behavior and peer support needs between men and women may play a role:

*...men maybe don’t talk to each other in the same way, like “Hey, I heard about this,” as we women do.*

[Mother of one (5.5 mo), ID3]

The effectiveness of the PICNIC peer model hinged around engendering a feeling of safety for participants. The combination of shared life stage (parenting) and exposure (PICNIC) meant participants were willing to share vulnerabilities, which then perpetuated deeper connection and a more meaningful experience. The model could require adaptation to be as effective for men and for different cultural groups.

#### Theme 3: Access and Enhancement

The theme of access and enhancement related to the suitability of accessing the PICNIC content for participants and other parents. They reflected on and compared their experience of accessing PICNIC content with previous experiences and considered other parents who may have more challenges accessing and engaging with PICNIC.

The live, online format was considered convenient, as parents enjoyed the social interaction while also easily participating from their home environment, with the capability to “mute themselves” when needing to care for their children. However, it was also posed that in-person workshops would be more “fun,” with the child health center suggested as a venue. Physical meetings were thought to boost discussions among parents and may also help increase engagement in the online Facebook group afterward:

*Of course, it helps if you can do it digitally,...you open the door for more people to participate. At the same time, I do feel that when you meet in person, it’s a bit easier to really be present in the moment and take in all the information. So there’s a bit of ambivalence in my answer, but in any case, some form of real-time workshop.*

[Mother of one (6 mo), ID8]

Recorded workshops or material were also discussed, and several parents highlighted the benefits of this approach, such as the ability to view it anytime, pause, and relisten, and to share it with their partner. Some also acknowledged the efficiency and potential for wider reach compared to live meetings. On the other hand, it would limit social interaction and viewing material may not be prioritized when it is not scheduled:

*If it’s a lecture you can watch anytime, it’s easy to say, “Yeah, we don’t have time tonight, we’ll do it tomorrow,” and then forget about it.*

[Mother of one (7 mo), ID7]

Overall, parents identified both advantages and disadvantages of different workshop delivery modalities, primarily related to ease of access. Some suggested having a range of options to accommodate differing needs, while others suggested a hybrid format. Parents also emphasized that the quality of a workshop largely depends on who leads it, regardless of the delivery method.

The accessibility of simple Facebook posts that reinforced workshop content was appreciated. Posts appeared in parents’ feeds and functioned as friendly reminders reinforcing PICNIC feeding messages. To boost engagement, creative content ideas and the combined use of multiple social media platforms were proposed:

*To have it appear in your daily scrolling, and then very simple things like “Think about this” or “Good things to know” or something. I think it’s a format that’s incredibly helpful, and especially for parents with small children, it can be so easily accessible*.

[Mother of one (7.5 mo), ID2]

As a hosting platform, Facebook was perceived by most parents as accessible and appropriate because of its closed group format with moderated content. However, some parents noted that Facebook use is lower among younger parents and therefore suggested other options be explored. Some also emphasized that because not everyone uses social media, emails with the same content should be included as a complementary feature.

Interviewees felt PICNIC’s focus on *how* to feed children was relevant across diverse cultures and family situations, and integrating it into standard CHS care was seen as a way to enhance credibility and accessibility. However, reaching and engaging different parents could be a challenge, and adaptations would be needed. Suggestions included translating and culturally adapting content for non-Swedish-speaking parents, and addressing other potential barriers such as low education, intellectual disabilities, and limited social media use:

*I think the content itself...it’s not too difficult for someone with only an upper secondary education, I think, or with a different cultural background. But the question is how you reach people.*

[Mother of two (youngest 6 mo), ID11]

To improve comprehension and engagement, alternative communication channels and tailored content, such as topic-specific workshops or question-and-answer sessions, were proposed. Parents expressed a desire for more practical meal tips, and one asked specifically for advice for a child with multiple allergies, noting limited guidance from CHS:

*Our son is allergic to pretty much everything—milk, eggs, nuts...and it’s really hard to find information and recipes and such, food-wise.*

[Mother of one (12 mo), ID9]

One mother described herself as the “food encyclopedia” at home and said she would have appreciated it if her partner had received the information directly. However, while many wished fathers would be more engaged in learning about responsive child feeding, they also recognized that it may be challenging to reach them with an intervention like PICNIC. They emphasized that the intervention probably needs to be “dad focused,” with information tailored specifically to fathers and their role. Some suggested more active recruitment through CHS but also at places where fathers tend to meet during parental leave:

*Maybe information from CHS*—*even if the mother goes [to CHS visits], she can bring the information home to the father*—*or open preschools [a free drop-in centre for parents and young children], where more fathers might be when children are a bit older...*

[Mother of one (12 mo), ID9]

A father who participated in the project highlighted the underrepresentation of fathers in such interventions and also suggested open preschools could be a way to engage them. During his parental leave, when his daughter was aged 10 to 18 months, he felt strongly responsible for her eating. However, he reported a lack of guidance and that other fathers would sometimes choose convenient “safe” options to avoid making mistakes:

*I’ve also noticed with other dads, yeah, it’s not so easy to find information...and if you want to be sure, you just grab a baby food pouch—then it can’t go wrong. Or things like that*.

[Father of one (21 mo), ID13]

Accessibility of accurate, relatable information, facilitators, and peer support was considered a key success factor in this pilot intervention. To enhance access, parents suggested using hybrid (in-person and online) delivery options or simulating characteristics of in-person participation as much as possible in the online environment. To effectively facilitate on-sharing of PICNIC content, social media platforms need to have suitable functionality and popularity with participants and prospective participant cohorts.

### Synthesized Data

In Table 3, key results from the quantitative and qualitative data are compared side-by-side based on framework domains reflected by the aims of the study: to understand feasibility, acceptability, appropriateness, and perceived impact of the PICNIC program.

**Table 3.** Quantitative and qualitative data integrated and compared based on the sequential mixed method study aims.

Domain	Quantitative	Qualitative	For further exploration
Feasibility (logistics)	Use of online and social media communications High workshop interest, enrollment, and participation High retention to follow-up Social media content feasible	All aspects highly feasible for mothers Peer education model feasible with PICNIC^a^ structure and format (workshop, closed social media group, and supporting resources)	PICNIC peer-to-peer education and support model would benefit from further exploration PICNIC scope requires clear boundaries explained (behavioral focus, can triage but not treat sensory and complex child feeding issues) Scalability of face-to-face model may limit feasibility
Acceptability (subjective, a good social fit)	High acceptability for parents of first child or with more than one child Relatively high “silent” engagement in the closed Facebook group, with a higher number of post views in user-created posts (47 views per post) than PICNIC social media posts (38 views per post) 93% would have continued in Facebook group 77% had shared with partner >50% had shared with friend or family Content easy to absorb and apply in practice	Highly acceptable to mothers, potentially acceptable to fathers, with modification Facebook group and website highly acceptable alternative to current information-seeking approaches Sense of solidarity and community Nonjudgmental tone supported engagement Social media platform acceptable to active sharers and observers, especially with known contributors (other PICNIC parents) and expert moderators Acceptability of PICNIC participation, engagement for fathers and culturally diverse groups requires further exploration Facilitator’s approach and demeanor valued	To optimize social media closed group engagement and access, parents need to feel secure (in platform and group) Adaptations needed to be feasible for culturally diverse groups Need for “dad focused” arm of PICNIC—they may have different ways of consuming and engaging with content and different mindsets about feeding
Appropriateness (suitable, fit for context, objective)	Complemented or supplemented child feeding support from child health services Appropriate to promote peer and family sharing of child feeding information or content 3‐4 months most appropriate child age for parent to commence PICNIC	Expertise of facilitator Application of responsive feeding practices Invitation to PICNIC around the time of introducing infant to solid food considered most appropriate Responsive feeding principles appropriate across cultural and gender contexts, but different approaches needed to reach different target groups	Consider separate groups for first-time parents and those with more than one child (or shared groups for peer learning)? Adaptations needed to be appropriate for fathers and culturally diverse groups
Perceived impact (outcomes, what happened as a result of involvement)	Significant increase in parental self-efficacy score from baseline to follow-up Self-reported increased practical skills for managing mealtimes	Increased feeding efficacy reported “Safe space” to share decreased stress and burden of child feeding Information from someone with lived experience perceived as trustworthy and impactful	Further research should measure child feeding, child diet quality, parental efficacy, and parenting-related anxiety outcomes over more than 12 months

a PICNIC: Parents In Child Nutrition Informing Community.

## Discussion

### Principal Findings

This study confirmed that the PICNIC program is feasible, acceptable, and appropriate and has potential for high impact in Sweden. Overall, parents participating in this pilot study reported improved confidence in their parental role regarding child feeding from participating in the project, and PSE also improved significantly from baseline to follow-up. In interviews, parents described how the advice shared in PICNIC had made a difference in their family meal situations. This, together with the evidence-based content and inclusive facilitation, encouraged parents to want to share the information with others. The focus on responsive feeding was considered applicable for “everyone.” Interviewed parents also identified the need and opportunities to improve reach to specific, underserved groups of parents and ways to optimize PICNIC program delivery. The intervention filled a gap complementing the support received from CHS and providing a sense of community and opportunity to share and recognize oneself in others.

### Comparison With Prior Work

This mixed methods pilot study indicated that the PICNIC program format is feasible and scalable, with adaptations needed for it to be acceptable and appropriate for fathers and culturally diverse groups. Consistent with qualitative analysis of the culturally adapted Healthy Beginnings program, feasibility and acceptability for culturally diverse groups depend on the availability of in-language resources and access to bicultural health professionals or support workers [38]. Language barriers between parents and health professionals can pose communication challenges [39], and Swedish CHS nurses have described a need for more cultural competence training and knowledge [40]. In the digital space, learnings from cultural adaptations of the MINISTOP mobile app in Sweden also showed that translation alone is not sufficient; literacy needs must also be addressed, for instance by providing video or audio formats [41].

Although this pilot study was not powered to assess intervention effectiveness, we observed a statistically significant improvement in PSE from baseline to follow-up. Both the baseline PSE level (mean 7.7, SD 1.5, among 42 complete cases) and the observed increase (0.48 points on a 0‐10 scale) were comparable to findings from the Swedish MINISTOP 2.0 trial, which used the same PSE item [32]. These findings suggest that the PSE item showed sufficient variability in our target population, with no indication of a ceiling effect, and was sensitive to change. This warrants further investigation in a well-powered interventional randomized controlled trial using validated PSE instrument(s).

The principles of responsive feeding form the basis of the PICNIC program and were considered feasible, acceptable, appropriate, and impactful in this pilot study. Participants expressed that responsive feeding is powerful and applicable across child ages and stages and across diverse cultural and gender contexts [6,42]. Although fathers’ feeding practices have been reported as modifiable when their partner participates in a child feeding intervention [43], direct involvement is likely to enhance engagement and peer sharing with other fathers. However, different approaches were considered necessary to reach fathers and other underserved groups, and examples of responsive feeding practices may need adaptation to pique interest and identification with the concepts [42]. For example, fathers may have a need for more organizational and practical support [44] or may engage more if they understand how influential their feeding practices are on their child’s health outcomes [45]. Individual peer-to-peer support from a “father mentor” trained in the PICNIC model could also be explored as an approach to specifically engage and support fathers. Similarly, concepts such as food security may resonate with parents from different cultural contexts [6].

The PICNIC program requires a highly developed and specific facilitation skill set, including expertise in both responsive feeding and the ability to apply the principles to meet the needs of a wide range of prospective participants. It was clear in this pilot study that this needs to be balanced with an empathic and approachable facilitation style. This finding is consistent with previous research, with a systematic review highlighting the importance of practitioners being nonjudgmental [46].

This pilot study showed that parents engage with PICNIC to meet their own needs in feeding children and that their altruistic intentions to support and educate other parents evolve as their own confidence grows and they want to share their learnings and experiences. This phenomenon mirrors the qualitative findings from PICNIC implementation in Australia, with delineation between peer educators’ and education recipients’ roles considered unrealistic because a high proportion of peer education recipients subsequently became peer educators. As a result, the “educator” role was therefore reframed toward a “champion” model, in which all parents exposed to the PICNIC intervention became “PICNIC parents” [26]. The impact of peer-to-peer support on recipients’ child feeding, along with the concept of a dual parent-peer role and the value of peer education versus peer support, requires further exploration in a larger trial.

To be sustainable and impactful, the PICNIC peer education model needs clear boundaries and referral pathways. The initial workshop provides an opportunity to discuss scenarios where PICNIC content is adequate and what to do if parents inquire about topics that are out of scope for parent-to-parent peer support, for example, sensory and complex child feeding issues. For enduring feasibility, PICNIC needs to optimize reach without overstretching resources, be clear about scope, and ensure facilitators are well trained with a combination of expertise and empathy, and that social media content and the website or other online information platforms are maintained.

The acceptability and appropriateness of the PICNIC pilot program were evidenced by high interest and uptake, high engagement, and enthusiastic quantitative and qualitative feedback. The format of involving an educational workshop to “meet and greet” and be “on the same page,” followed by ongoing incremental “drip feeding” of content, served a dual purpose. This format is also consistent with the educational concept of balancing reinforcement and extension of learning, without information overload or message fatigue [47]. In parallel, familiarity and trust were built between participants and with the facilitator, which served to create the “safe space” for sharing. Meanwhile, participants in the present study identified pros and cons with in-person versus online and suggested recorded or self-directed learning in addition to interactive live sessions. Thus, alternative modes of delivery should be explored when PICNIC is further adapted for the Swedish context to optimize reach to a diverse group of parents while using resources effectively.

It was notable that participants felt more comfortable sharing in person or with close peers, whereas sharing through liking or on-sharing social media posts was less common. This reluctance among some parents to like and share content may reflect their insecurity about feeling vulnerable in their parenting role in a wider, less familiar forum or a limited awareness that liking and sharing could be a way to on-share the information that resonated with them. Previous research has shown that “nonclickers” on social media can still be active content viewers and that personality and perceived attitudes toward clicking and on-sharing can influence decisions to do so [48]. Finding a balance between broader peer sharing while maintaining the sense of a “safe space” is a challenge for the PICNIC program as it expands to more parent groups [49].

Anticipatory guidance is a strategy used effectively in the original Australian PICNIC program to match content to the learning needs of participants. As children’s eating behaviors evolve rapidly with their stage of development, anticipating what they will likely experience in the next stage is both a science and an art [50]. The age of the children was described as a key factor for how well the parents related to the content in PICNIC, with early intervention around the time of introducing solids considered ideal. Relatable content that they could recognize themselves in was most valued, and some parents therefore suggested making the intervention more age specific. This suggestion was counterbalanced by feedback highlighting the value of input from parents who had more than one child or had already experienced situations relevant to a child transitioning through that particular stage. To increase the acceptability and appropriateness of content across child ages and stages, while remaining logistically feasible, PICNIC programming needs to consider and incorporate these concepts and expectations into planning, implementation, and continuous evaluation.

### Strengths and Limitations

The strengths of this study included the mixed methods approach in which qualitative data corroborated and contextualized the quantitative results. Maximum variation sampling was used for the interviews to capture a broad range of participant perspectives and experiences, where 15 interviews were considered sufficient for ensuring data richness and saturation. The trustworthiness of the study was also strengthened by investigator triangulation, in which 2 investigators at different professional levels independently coded the qualitative data. The other coauthors then discussed and provided feedback on the results in an iterative process, revisiting the original data when needed. As this was a pilot study, baseline recruitment continued until at least 40 participants had enrolled and were scheduled for 1 of the 10 PICNIC workshops offered. This was considered adequate and feasible for the nature of the study, but not large enough to measure objective outcomes. Nevertheless, despite the small sample size, we observed a statistically significant improvement in PSE from baseline to follow-up. We did not quantitatively measure intervention effectiveness, such as parents’ child feeding practices or children’s eating behaviors; this should be investigated further in a well-powered randomized controlled trial.

Furthermore, most parents who participated in this study were female and highly educated. This pattern is commonly seen in this field of research and is a limitation that may influence the transferability of findings to other parent groups. However, it also indicates which parents are most likely to engage voluntarily in PICNIC, suggesting that others may require more active outreach. At the same time, it was surprising that as many as 43% (20/47) of our study participants had not been invited to a parent support group at their child health center. Such groups should be offered as universal CHS support [16], but national statistics from 2023 to 2025 show that only 20% to 30% of children have parents attending [51]. This indicates that further efforts are needed to expand group health care in Sweden and address barriers to parent participation. Previous qualitative research shows that mothers often feel isolated during the first year of parenthood and express a need to share their experiences and to feel supported, reassured, and understood [44]. Parents who cannot attend physical CHS group meetings may therefore benefit from PICNIC’s online peer support. Meanwhile, Swedish CHS follows the principle of proportionate universalism, aiming to provide equitable care by offering targeted support to families with greater needs. This should guide prioritization of target groups, especially if resources are limited.

### Conclusions

This pilot study indicated that Swedish parents of young children found the PICNIC peer education model feasible, acceptable, and appropriate for supporting parents in responsive child feeding. Engaging with the intervention resulted in parents feeling more confident in their parental role and in the child feeding process. Recognizing their own experiences in other parents’ questions within a nonjudgmental “safe space” felt reassuring. The parents highly valued the direct access to expert advice and opportunities to interact with other parents, while also seeing value in recorded content. A large-scale effectiveness-implementation trial will evaluate whether “PICNIC Sweden” can improve feeding practices and children’s eating behaviors. The planned study will also determine how the program can be realistically scaled within available resources, CHS settings, and conditions.

## Supplementary material

10.2196/93565 Multimedia Appendix 1 Interview guide.

10.2196/93565 Checklist 1 COREQ and GRAMMS checklists.

## Abbreviations

CHS: child health services
COREQ: Consolidated Criteria for Reporting Qualitative Research
GRAMMS: Good Reporting of a Mixed Methods Study
PICNIC: Parents in Child Nutrition Informing Community
PSE: parental self-efficacy
PSEPAD: Parental Self-Efficacy for Promoting Healthy Physical Activity and Dietary Behaviors in Children
