# Supplementary material for: Parents’ Experiences From Participating in an Online Peer Support Child Feeding Intervention: The “PICNIC Sweden” Explanatory Sequential Mixed Methods Feasibility Study
Source: JMIR Pediatr Parent. 2026 Sep 22;9:e93565. doi: 10.2196/93565 (PMC13597015; doi:10.2196/93565)
Supplement: Supplementary file 1 [file pediatrics-v9-e93565-s001.pdf]

Interview guide

Introduction included information on the aim of the interview, anonymity in data analyses and presentation, expected time of interview (no more than 60 min), that no answers are right or wrong but all feedback is welcome, the semi-structured format meaning the participant can also bring up additional issues/feedback, and a reminder that the interview is being audio recorded.

Introductory questions:

1. How have you experienced being part of the pilot study?
2. Can you tell me what, or which parts of the PICNIC project, you found most rewarding?

Suitability and acceptability

3. In PICNIC, you received information and advice — how useful do you think these have been?
4. PICNIC uses a workshop, social media, and a website to support parents on topics related to children and food. This information can then be shared by PICNIC participants with others. What do you think about this setup?
5. The survey asked about how people think the workshop should be delivered. What are your thoughts on this?
6. Do you have any thoughts about groups of parents for whom PICNIC may not be suitable?
   - Why do you think these groups are not a good fit for PICNIC?
   - How do you think PICNIC could better reach fathers, or other individuals who play a central role in the child's mealtime situations?

Sharing of information

7. Have you shared the information from PICNIC with others? (e.g. friends, family, other parent)
   - How did you feel the information was received by them?

The PICNIC workshop

8. What did you take away from the workshop? Can you describe how you felt?
9. What advice would you give the PICNIC team to improve and develop this workshop?

Facebook

10. In what way have you engaged with PICNIC on social media? (e.g., read posts, liked, commented, or asked questions)
11. How did you perceive the texts and images shared through the Facebook posts?
    - What do you think contributes to parents liking or commenting on posts?

Perceived impact

12. How has PICNIC influenced you in your role as a parent?
13. What is the most important thing you have gained from participating in PICNIC?

Closing questions

14. What are your thoughts on offering PICNIC on a larger scale in Sweden?
15. Finally, what do you consider the most important aspect to take forward in our ongoing work with PICNIC?
16. Is there anything I've missed asking, or do you have any other thoughts or comments you'd like to bring up?
